# Cancer-associated fibroblast-derived PAI-1 promotes lymphatic metastasis via the induction of EndoMT in lymphatic endothelial cells

**DOI:** 10.1186/s13046-023-02714-0

**Published:** 2023-07-06

**Authors:** Wen-Fei Wei, Hui-Ling Zhou, Pei-Yu Chen, Xiao-Lan Huang, Long Huang, Luo-Jiao Liang, Chu-Hong Guo, Chen-Fei Zhou, Lan Yu, Liang-Sheng Fan, Wei Wang

**Affiliations:** 1 Department of Gynaecology, Zhuhai People’s Hospital (Zhuhai Hospital Affiliated With Jinan University), Zhuhai, 519000 Guangdong China; 2 Department of Obstetrics and Gynaecology, the First Affiliated Hospital of Guangzhou Medical University, Guangzhou, 510120 Guangdong China; 3 Department of Obstetrics and Gynaecology, Nanfang Hospital, Southern Medical University, Guangzhou, 510515 China; 4 Department of Urology, Zhuhai People’s Hospital (Zhuhai Hospital Affiliated With Jinan University), Zhuhai, 519000 Guangdong China; 5 Department of Gynecology, Guangdong Provincial People’s Hospital, Guangdong Academy of Medical Sciences, Guangzhou, 510080 Guangdong China; 6 Department of Gynecologic Oncology, State Key Laboratory of Oncology in South China, Collaborative Innovation Center for Cancer Medicine, Sun Yat-Sen University Cancer Center, Guangzhou, 510060 Guangdong China

**Keywords:** Cancer-associated fibroblast, Endothelial-mesenchymal transition, Cervical squamous cell carcinoma, Lymphatic metastasis, PAI-1

## Abstract

**Background:**

Endothelial-mesenchymal transition (EndoMT) is an emerging adaptive process that modulates lymphatic endothelial function to drive aberrant lymphatic vascularization in the tumour microenvironment (TME); however, the molecular determinants that govern the functional role of EndoMT remain unclear. Here, we show that cancer-associated fibroblast (CAF)-derived PAI-1 promoted the EndoMT of lymphatic endothelial cells (LECs) in cervical squamous cell carcinoma (CSCC).

**Methods:**

Immunofluorescent staining of α-SMA, LYVE-1 and DAPI were examined in primary tumour samples obtained from 57 CSCC patients. Assessment of cytokines secreted by CAFs and normal fibroblasts (NFs) was performed using human cytokine antibody arrays. The phenotype of EndoMT in lymphatic endothelial cells (LECs), gene expression levels, protein secretion and activity of signaling pathways were measured by real-time RT-PCR, ELISA or western blotting. The function of lymphatic endothelial monolayers was examined by transwell, tube formation assay, transendothelial migration assay in vitro. Lymphatic metastasis was measured using popliteal lymph node metastasis model. Furthermore, association between PAI-1 expression and EndoMT in CSCC was analyzed by immunohistochemistry. The Cancer Genome Atlas (TCGA) databases was used to assess the association of PAI-1 with survival rate in CSCC.

**Results:**

CAF-derived PAI-1 promoted the EndoMT of LECs in CSCC. LECs undergoing EndoMT could initiate tumour neolymphangiogenesis that facilitated cancer cell intravasation/extravasation, which in turn promoted lymphatic metastasis in CSCC. Mechanistically, PAI-1 activated the AKT/ERK1/2 pathways by directly interacting with low-density lipoprotein receptor-related protein (LRP1), thereby leading to elevated EndoMT activity in LECs. Blockade of PAI-1 or inhibition of LRP1/AKT/ERK1/2 abrogated EndoMT and consequently attenuated CAF-induced tumour neolymphangiogenesis. Furthermore, clinical data revealed that increased PAI-1 levels positively correlated with EndoMT activity and poor prognosis in CSCC patients.

**Conclusion:**

Our data indicate that CAF-derived PAI-1 acts as an important neolymphangiogenesis-initiating molecular during CSCC progression through modulating the EndoMT of LECs, resulting in promotion of metastasis ability in primary site. PAI-1 could serve as an effective prognostic biomarker and therapeutic target for CSCC metastasis.

**Supplementary Information:**

The online version contains supplementary material available at 10.1186/s13046-023-02714-0.

## Introduction

Cancer progression has been considered a consequence of the evolving crosstalk between different cell types within the tumour microenvironment (TME) [1, 2]. The remodelling of regional lymphatic vessels (LVs) is associated with enhanced malignant progression and poor outcome in solid tumours, including cervical squamous cell carcinoma (CSCC) [3]. In addition to offering physical routes for metastatic spread [4], tumour-associated LVs have emerged as active players in the modulation of prometastatic responses [5]. Therefore, metastatic and primary tumour progression can be affected by manipulating tumour-associated lymphatic remodelling [6]. Endothelial–mesenchymal transition (EndoMT) was recently reported to be a hallmark of lymphatic remodelling [7]. During EndoMT, lymphatic endothelial cells (LECs) undergo a phenotype switch to generate elongated, spindle-shaped mesenchymal-like cells that are highly migratory and invasive but lack cellular junctions [8, 9]. These changes promote LEC dysfunction to induce the EndoMT-related abnormal vasculature, which shares high similarities with tumour neolymphangiogenesis and reinforces the important adaptive role of EndoMT in the TME that favours tumour dissemination [2, 10]. An improved understanding of EndoMT could therefore uncover a novel mechanism of lymphatic metastasis. However, little is currently known about the triggers and functional significance of EndoMT in CSCC.

Cells or cytokines within the regional microenvironment influence the biological properties of LECs [11]. Cancer-associated fibroblasts (CAFs) are activated fibroblasts and the most abundant stromal component in the TME [12]. CAFs produce paracrine growth factors, proteolytic enzymes, and ECM components and contribute to disrupting the biochemical and biomechanical homeostasis of the TME [13]. These perturbations are sensed by other TME components, which ultimately affect their behaviour, including the promotion of EndoMT in LECs [13]. In our previous study, the presence of CAFs was associated with LEC dysfunction in primary tumours, implying an active role for these fibroblasts in tumour-associated lymphatic remodelling [14]. Therefore, the interplay between CAFs and LECs in promoting lymphatic metastasis warrants further investigation.

In this study, we observed EndoMT of LECs in lymphatic metastatic CSCC and determined the relevance of this observation to CAFs. Our findings suggest that CAFs secrete PAI-1 to induce EndoMT by activating low-density lipoprotein receptor-related protein (LRP1)/AKT/ERK1/2 signalling. However, inhibition of this axis abrogated EndoMT and alleviated the EndoMT-related abnormal lymphatic vasculature induced by CAFs. These results suggest that the EndoMT of LECs might represent a novel mechanism for CAF-dependent lymphatic metastasis and a potential target for the prevention and treatment of CSCC metastasis.

## Materials and methods

### Clinical specimens

All cervical specimens were obtained from patients who voluntarily consented to study participation at the Department of Gynecological Oncology of The First Affiliated Hospital of Guangzhou Medical University (Guangzhou, China). This study was approved by the Institutional Research Ethics Committee and compliant with the principles of the Declaration of Helsinki.

The paraffin-embedded cervical specimens, including 37 CSCC cases without LNM and 20 CSCC cases with LNM, were subjected to immunohistochemical and immunofluorescence analyses. Fibroblasts were isolated from fresh samples, including 8 normal cervical tissue samples from multiple hysteroma patients that underwent hysterectomy and 8 cervical cancer tissue samples from CSCC patients who underwent abdominal radical hysterectomy without prior radiotherapy and chemotherapy.

### Cell culture and transfection

The human cervical cancer cell line SiHa was purchased from American Type Culture Collection (ATCC, Manassas, VA, USA) and cultured according to the supplier’s guidelines. HDLECs were purchased from ScienCell Research Laboratories (Carlsbad, CA, USA) and cultured in endothelial cell medium (ECM; ScienCell) with 5% FBS (Gibco, Invitrogen, Carlsbad, CA, USA). SiHa cells were transfected with lenti-mCherry; cells stably expressing mCherry were selected for further experiments. HDLECs were transfected with lenti-GFP, and cells stably expressing GFP were selected.

### Isolation and identification of CAFs and NFs from fresh CSCC samples and normal cervical samples

CAFs and NFs were isolated and identified as previously described [15]. Primary fibroblasts at no more than 10 passages were used for the experiments.

### Preparation of CM

CM was prepared as previously described [15]. Briefly, fibroblasts were grown to ~ 80% confluence in growth culture medium. The cells were washed and incubated in ECM (ScienCell) with all supplements and 2% FBS (Gibco) at 37 °C for 48 h. The fibroblast CM was subsequently harvested, centrifuged at 2,000 × *g* for 5 min, and filtered using 0.2 μm membrane syringe filters to eliminate cell debris. The cleared CM was collected and added to the endothelial cell monolayer for in vitro or in vivo permeability assays.

### RNA extraction and qRT-PCR

RNA was extracted from cells using TRIzol (Invitrogen, California, USA). qRT-PCR was performed as previously described [16]. The primer sequences are shown in Table S2. The expression level of each mRNA was normalized to that of GAPDH.

### Western blotting

Western blotting assays were performed as previously described [16]. The primary antibodies were as follows: anti-vimentin, anti-FAP, anti-αSMA, anti-LYVE-1, and anti-PAI-1 (Abcam, Cambridge, MA, USA); anti-phospho-AKT (Ser 473), anti-AKT, anti-phospho-ERK1/2 (T202/Y204), anti-ERK1/2, anti-p38, anti-phospho-p38, anti-JNK, anti-phospho-JNK, anti-VE-cadherin, anti-phospho-VE-cadherin (Y685) and anti-GAPDH (CST, Massachusetts, USA). The secondary antibodies were horseradish peroxidase-conjugated anti-rabbit or anti-mouse immunoglobulin-G antibody (Abcam, Cambridge, MA, USA).

### Human cytokine array

When the fibroblasts reached ~ 80% confluence, the medium was replaced with serum-free medium. Forty-eight hours later, the medium was harvested and analysed with a human cytokine antibody array (RayBio Human Cytokine antibody arrays QAH-CYT9 and AAH-CYT-G9) following the manufacturer’s instructions.

### ELISA

The concentration of PAI-1 in CAF/NF-CM was determined by commercially available PAI-1 ELISA kits (eBioscience, San Diego, USA). ELISAs were performed according to the manufacturer’s protocol.

### Immunohistochemistry

Tissue sections were subjected to immunohistochemical analysis as described previously [17]. The primary antibodies were as follows: anti-PAI-1 (Abcam), anti-αSMA (Abcam), and anti-LYVE-1 (Abcam). The secondary antibody was horseradish peroxidase-conjugated anti-rabbit immunoglobulin-G antibody (Abcam).

### Staining assessment

The tissue sections stained by immunohistochemistry and in situ hybridization were reviewed and scored independently by two pathologists. For the semiquantitative evaluation of protein levels in tissue, an immunoreactivity scoring system (the H-score) was used as previously described [17]. An H score ≤ 2.0 indicated a low protein level, and an H score > 2.0 indicated a high protein level.

### Multiplexed immunofluorescence assay

Multiplexed immunofluorescence was performed using an Opal 4-Color Fluorescence IHC Kit (PerkinElmer, Waltham, MA, USA) according to the manufacturer’s protocol with the following primary antibodies: anti-α-SMA, anti-LYVE-1, anti-PAI-1, and anti-VE-cadherin (Abcam).

After deparaffinization, the sections were microwaved in antigen retrieval buffer for 45 s at 100 °C; then, the temperature was reduced by 10–20% for 15 min to prevent boiling. The sections were washed, blocked at room temperature for 10 min, and incubated with primary antibody. The slides were then incubated for 10 min at room temperature with HRP-conjugated secondary antibody; subsequently, TSA working buffer containing Opal 520, Opal 570, Opal 650, and DAPI was used for signal amplification. After removing the primary and secondary antibodies by microwaving the slides, the same procedures were repeated with the next primary antibody and TSA working buffer. The fluorescence intensity of the protein signal was analysed using ZEN2.1/ZEN2 software (Carl Zeiss Microscopy GmbH). To calculate the mean vessel intensity, the sum of the pixel intensities per vessel was divided by the total vessel area (mm^2^). The mean vessel fluorescence intensity (MFI) from five images per specimen was computed and compared between groups.

### In vivo tube formation

HDLECs subjected to different treatments were mixed with Matrigel at a 1:1 ratio (BD Biosciences, San Jose, CA, USA), and 50 μl of the mixture was injected subcutaneously into female nude mice (5 weeks old). Matrigel mixed with medium served as a negative control. The Matrigel plugs were removed 15 days after implantation, paraffin-embedded and analysed by H&E staining. Tube morphogenesis was assessed using a Nikon upright microscope.

### Popliteal LNM model

Female nude mice (5 weeks old) were purchased from the Experimental Animal Centre, Southern Medical University (Guangzhou, China). All experimental procedures were approved by the Institutional Animal Care and Use Committee of Guangzhou Medical University. The popliteal lymphatic metastasis model was established in nude mice by inoculating the footpad with SiHa-mCherry cells. Tumour size (mm^3^) was measured twice a week and calculated by the formula Volume = (width)^2^ × length/2. When the footpad tumour reached 50 mm^3^, CM derived from CAFs or NFs was then randomly injected into the centre of the xenograft tumours twice a week. The mice were euthanized when the primary tumours were approximately 150 mm^3^. The number of metastases was tracked in live mice by optical imaging of mCherry using the In Vivo FX PRO system (Bruker, Billerica, MA, USA). The primary tumours were paraffin embedded and analysed for lymphoinvasion and EndoMT in LVs by IHC and immunofluorescence, respectively. The popliteal LNs were analysed for mCherry expression by IHC with an anti-mCherry antibody (Abcam). Positive LNs were identified by detecting mCherry staining under a Nikon upright microscope. The ratio of metastasis-positive to total dissected popliteal LNs was calculated.

### Transendothelial migration assay

A transendothelial migration assay was performed as described in our previous study [15]. The experiments were performed in triplicate, and the results represent three independent experiments.

### Public data analysis

Gene expression data were obtained from The Cancer Genome Atlas (TCGA) (https://cancergenome.nih.gov/). Kaplan–Meier survival analyses were conducted to compare outcomes based on periostin expression levels in 301 cervical cancer patients. Based on the median PAI-1 expression level (330,979.5462) in TCGA mRNA-Seq data, patients were classified into the PAI-1-high group (expression level ≥ 330,979.5462; 149 patients) and the PAI-1-low group (expression level < 330,979.5462; 152 patients).

### Statistical analysis

Statistical analysis was performed using SPSS v.20.0 software (SPSS Inc., Chicago, IL, USA). The data are expressed as the mean ± standard deviation (SD). One-way ANOVA was used for comparisons among groups, and the chi-squared test (χ^2^ test) was applied to categorical variables. Correlation analysis was performed using Spearman’s rank test. Differences were considered statistically significant at *p* < 0.05.

## Results

### The increasing number of CAF positively correlates with the upregulation of mesenchymal phenotype of LECs in CSCC

**Fig. 1:**
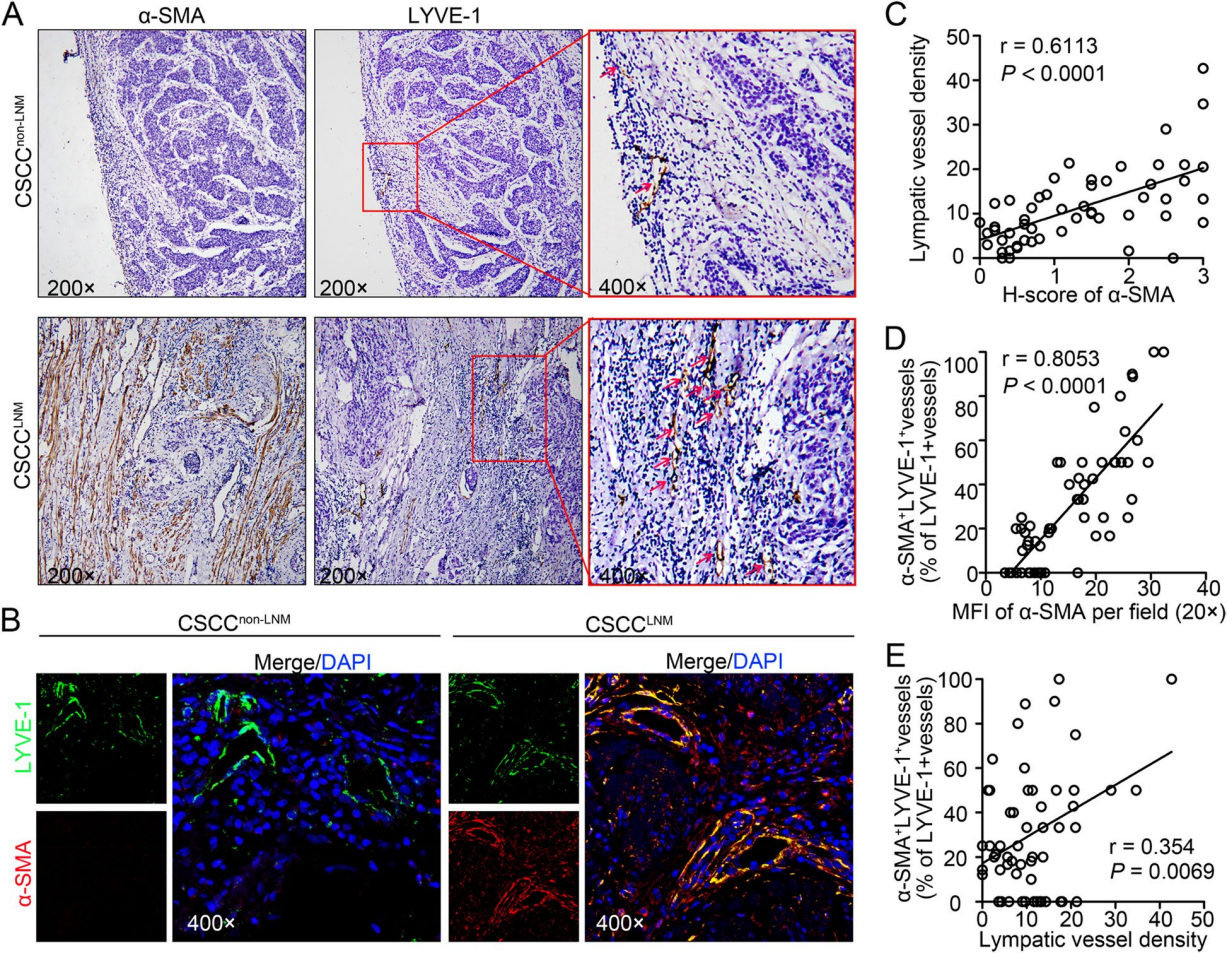
The increasing number of CAF positively correlates with the upregulation of mesenchymal phenotype of LECs in CSCC. **A** Staining for α-SMA (CAF marker) and LYVE-1 (lymphatic marker) in serial sections of CSCC specimens. The lymphatic vessels are indicated by red arrows. **B** Representative images of LYVE-1 (green) and α-SMA (red) fluorescence staining in CSCC (20 samples with LNM; 37 samples without LNM) under 400 × magnification. Blue indicates the nucleus. **C** Correlations between CAFs and lymphatic vessel density were analysed. **D** The correlation between α-SMA^+^LYVE-1^+^ vessels and CAFs (α-SMA marker). **E** The correlation between α-SMA^+^LYVE-1.^+^ vessels and lymphatic vessel density. *p < 0.05

It has been reported that CAFs can induce functional responses in LECs, and neolymphangiogenesis plays a critical role in promoting metastatic progression. Therefore, the expression of α-SMA (a CAF marker) and LYVE-1 (a lymphatic marker) in serial sections of human CSCC samples was analysed using immunohistochemistry to investigate whether CAFs are responsible for the generation of a rich lymphatic plexus in the TME of CSCC. As shown in Fig. 1A and C, higher CAF levels strongly correlate with the increment of lymphatic vessel density (LVD) (*r* = 0.6113, *P* < 0.0001). To determine the effect of CAFs on the plasticity of LVs in CSCC, 57 primary tumours (from 37 CSCC patients without lymph node metastasis (LNM) [CSCC^non−LNM^] and 20 with LNM [CSCC^LNM^]) were subjected to dual-label immunofluorescence for α-SMA and LYVE-1. We observed two distinct lymphatic patterns: LYVE-1^+^ vessels with positive expression of α-SMA (α-SMA^+^LVs) in CSCC^LNM^ and LYVE-1^+^ vessels without α-SMA expression in CSCC^non−LNM^. This phenomenon suggested that the EndoMT of LECs was prevalent in CSCC^LNM^. The clinical relevance of this finding was analysed by Pearson’s coefficient test, which identified a significant correlation between the ratio of α-SMA^+^LVs and the abundance of CAFs (*r* = 0.8053, *P* < 0.0001, Fig. 1D) and the LVD (*r* = 0.354, *P* = 0.0069, Fig. 1E). Collectively, these results suggest that the density of CAFs and LVs is increased in CSCC^LNM^, coinciding with increased expression of the mesenchymal phenotype in LECs.

### CAF-induced EndoMT promotes LV abnormalities in vitro

**Fig. 2:**
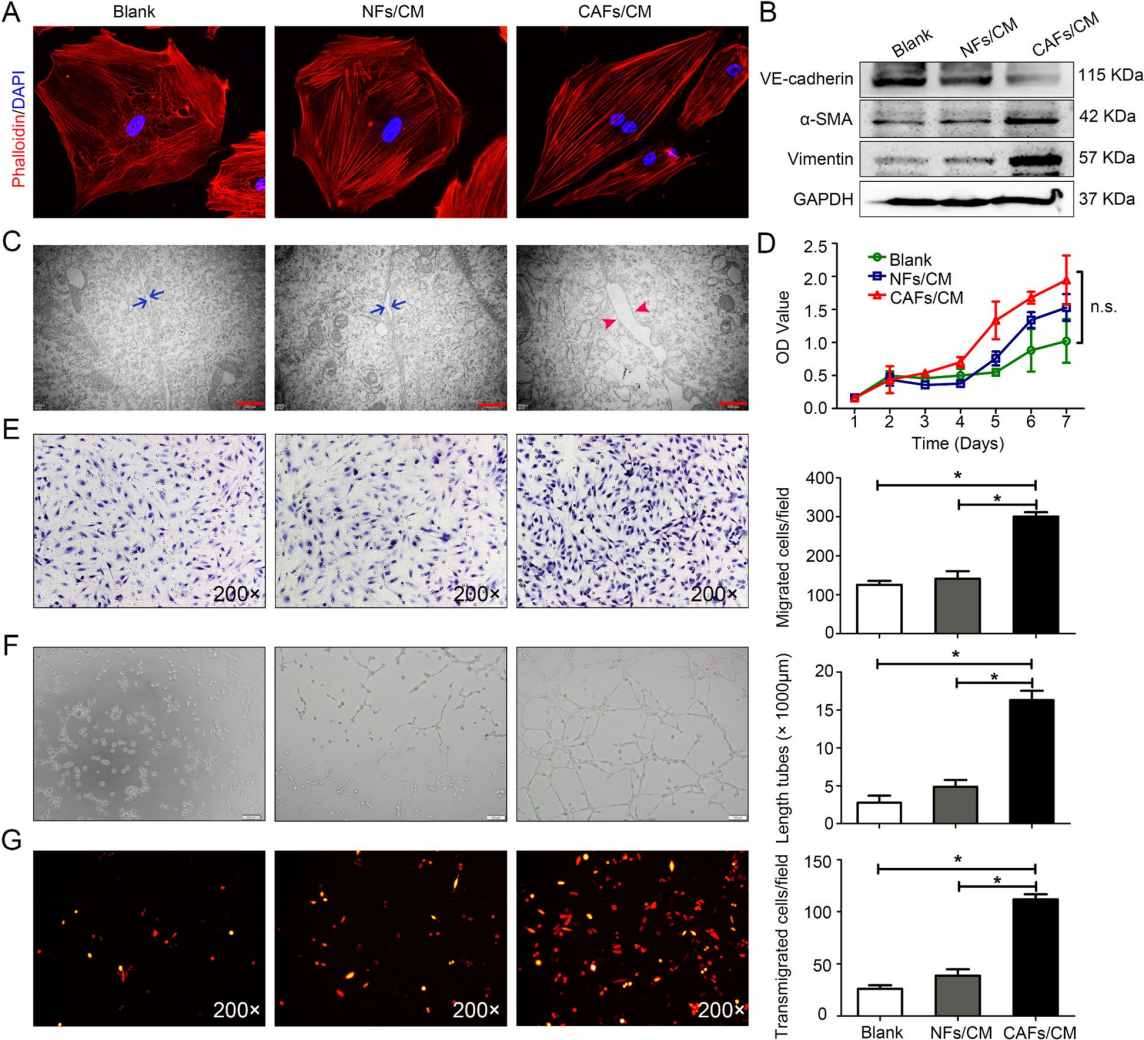
CAF-induced EndoMT promotes LV abnormalities in vitro. **A** The morphology of HDLECs pretreated with NF/CAF-CM was observed, and untreated HDLECs served as the blank group. Cytoskeletal F-actin was stained with rhodamine-phalloidin and viewed under a fluorescence microscope at 400 × magnification (lower panel). **B** Western blotting analysis of VE-cadherin, α-SMA and vimentin in CM-treated HDLECs. **C** Transmission electron microscopy images showing the lymphatic endothelial cell–cell junction integrity after treatment with control medium, NF-CM or CAF-CM (Blue arrows indicate intercellular gap of Blank and NF-CM group; Red arrows indicate intercellular gap of CAF-CM group, panels: 40,000 × magnification; scale bar: 500 nm). **D** Cell proliferation was measured by CCK8 assays. n.s., not statistically significant. **E** Representative micrographs (left panel) of Transwell assays using HDLECs pretreated with the indicated CM are shown. Scale bar, 50 μm. The average number of migrated cells per field was calculated (right panel). **F** Representative micrographs (left panel) of tube formation assays using HDLECs pretreated with the indicated CM. Scale bar, 100 μm. The average tube length per field was calculated. **G** Confluent HDLEC monolayers were treated as indicated for 24 h. SiHa-mCherry cells were seeded onto the monolayers for another 24 h, and the number of transmigrated SiHa-mCherry cells was quantified. Data are presented as the mean ± SD of three independent experiments. *P < 0.05

To further analyse the dynamic interaction between CAFs and LECs, we isolated normal fibroblasts (NFs) from normal cervical tissues and CAFs from CSCC tissues. Human dermal lymphatic endothelial cells (HDLECs) were treated with conditioned medium (CM) from CAFs (CAF-CM) or NFs (NF-CM). Analysis of the morphology of phalloidin-stained HDLECs showed that treatment with CAF-CM markedly altered the shape of HDLECs, with the morphology shifting from the characteristic cobblestone appearance to a fibroblast-like spindle shape (Fig. 2A). The Western blotting results showed that CAF-CM increased the levels of mesenchymal proteins, including vimentin and α-SMA, but decreased the expression of the endothelial-specific marker VE-cadherin (Fig. 2B). These results indicate that CAFs induce EndoMT in HDLECs.

To investigate EndoMT-related functional changes, we analysed HDLEC proliferation, migration and tube formation ability after the indicated treatments. The results showed that CAF-CM dramatically increased HDLEC migration (*P* < 0.05, Fig. 2E) and tube length (*P* < 0.05, Fig. 2F) relative to the other treatments. In contrast, HDLEC proliferation did not differ among groups (n.s. *P* > 0.05, Fig. 2D), suggesting that the cytoskeletal remodelling of LVs induced by CAF-CM, rather than the enhanced proliferation of HDLECs, evoked the difference in tube-formation ability.

EndoMT leads to a decrease in the endothelial adhesion molecule VE-cadherin, which may impair the integrity of intercellular barriers. We therefore evaluated the integrity of the LEC monolayer during EndoMT under CAF-CM treatment. Transmission electron microscopy revealed intact tight junctions between HDLECs in the untreated group and in the NF-CM-treated group. Conversely, the cell–cell junctions were disrupted, with increased space between adjacent cell membranes, in the CAF-CM-treated HDLEC monolayers (Fig. 2C). Next, a transendothelial migration assay was used to mimic the cancer cell intravasation/extravasation process in vitro to further determine the consequence of LEC barrier disruption during EndoMT. The transendothelial migration of SiHa-mCherry cells was significantly higher in the CAF-CM-treated group (*n* = 111.67 ± 5.03) than in the other groups (control group: *n* = 26.00 ± 3.60; NF-CM-treated group: *n* = 38.67 ± 6.11) (Fig. 2G, *P* < 0.05). Collectively, these results suggest that CAF-induced EndoMT not only promotes lymphangiogenesis in vitro but also facilitates tumour transendothelial migration by disrupting the integrity of the endothelial barrier.

### CAFs induce EndoMT and promote lymphangiogenesis in vivo

**Fig. 3:**
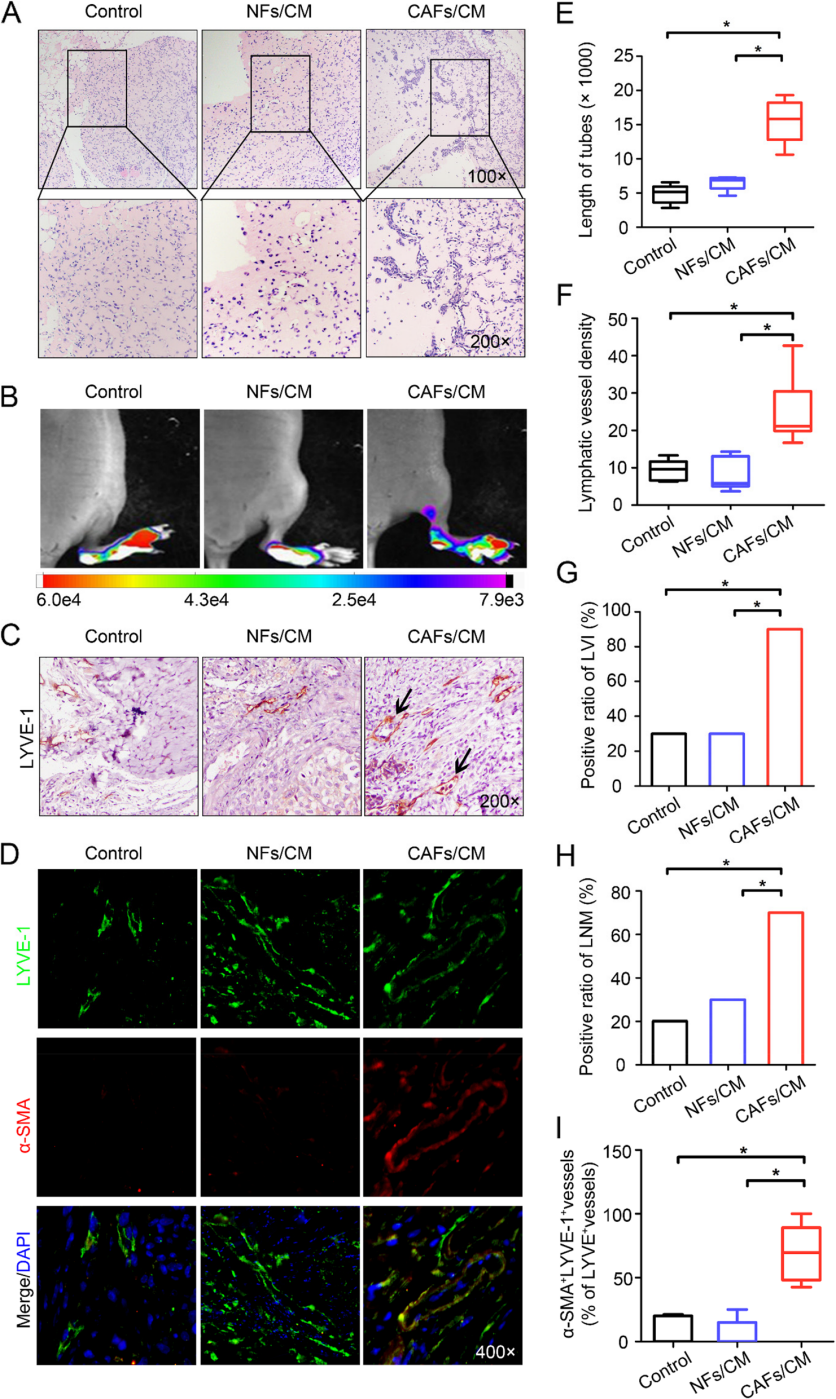
CAFs induce EndoMT and promote lymphangiogenesis in vivo. **A** Representative images showing tube formation in vivo (left panels: 100 × magnification; right panels: 400 × magnification). **B** In vivo fluorescence images of lymphatic metastasis (*n* = 10). **C** Staining of LYVE-1 in footpad tumours. Representative micrographs of positive staining are shown. Blank arrows indicate cancer cells that invaded the LVs. **D** Paraffin-embedded tumour sections from the experimental mice were stained with both anti-LYVE-1 (green) and anti-α-SMA (red) antibodies, and representative images are shown at 400 × magnification. **E** Statistical analysis showing the length of tube formation. The average tube length per field was calculated. **F** Statistical analysis of the LVD in footpad tumours. **G** Statistical analysis of lymphatic vessel invasion (LVI) in footpad tumours. **H** Ratio of metastasis-positive LNs to total dissected popliteal LNs in mice treated with the indicated CM. **I** Percentage of α-SMA^+^LYVE-1^+^ vessels among total LYVE-1.^+^ vessels in footpad tumours. *P < 0.05

To further demonstrate the in vivo effect of CAFs on HDLECs, Matrigel plugs containing HDLECs alone or HDLECs mixed with either CAF-CM or NF-CM were injected subcutaneously into female nude mice. Haematoxylin and eosin (H&E) staining analysis of the Matrigel sections showed that HDLECs mixed with CAF-CM formed significantly longer tubes [(15.56 ± 3.21) × 1000 μm] in vivo than HDLECs alone [(4.98 ± 1.35) × 1000 μm] or those mixed with NF-CM [(6.48 ± 1.01) × 1000 μm] (*P* < 0.05, Fig. 3A and E). We further inoculated mCherry-labelled SiHa cells into the footpads of mice. When the tumour reached 50 mm^3^, CAF-CM or NF-CM was then randomly injected into the centre of the xenograft tumours twice a week. After 4 weeks, the CAF-CM-treated group showed a significantly higher LVD (25.17 ± 8.29) (*P* < 0.05) (Fig. 3C and F) than the control (9.41 ± 2.53) or NF-CM-treated group (8.30 ± 4.22), accompanied by an increased ratio of lymphovascular space invasion (CAF-CM-treated group *vs.* NF-CM-treated groups *vs.* control group = 90% *vs.* 30% *vs.* 30%) (*P* < 0.05) (Fig. 3C and G). Additionally, an immunofluorescence assay of LVs in the CAF-CM-treated group revealed positive expression of α-SMA (Fig. 3D and I), verifying that EndoMT in LECs was promoted by CAFs in vivo. Consistent with the effect of enhancing the EndoMT activity of LECs, CAF-CM significantly promoted LNM in vivo (70%, 30% and 20% in the CAF-CM-treated, NF-CM-treated and control groups, respectively; *P* < 0.05) (Fig. 3B and H, Table S1). Collectively, these results suggest that CAFs increase the metastatic potential by enhancing EndoMT activity in LECs to induce abnormally extensive LV in the host.

### PAI-1 derived from CAFs is required to induce EndoMT in LECs

**Fig. 4:**
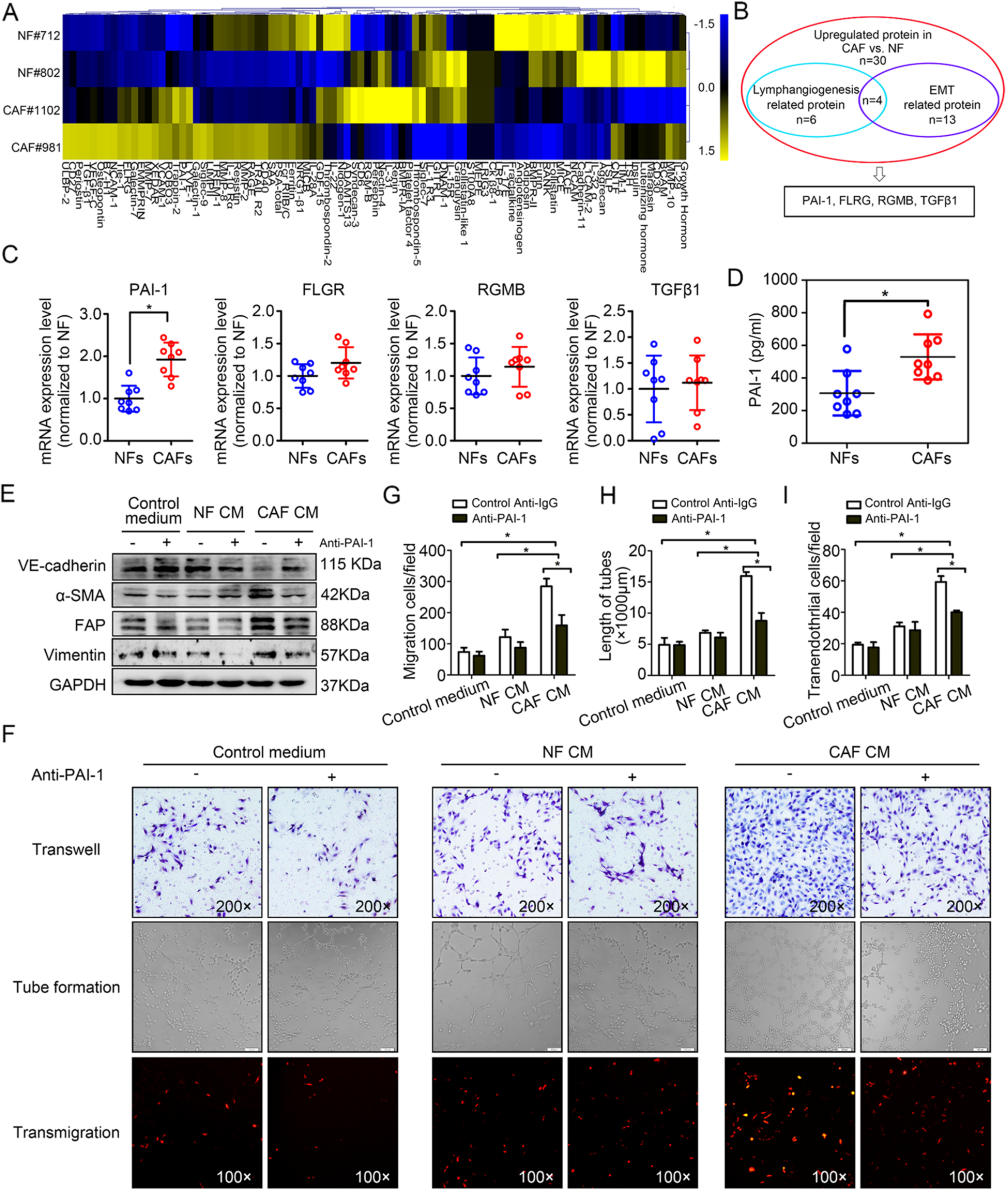
PAI-1 derived from CAFs is required to induce EndoMT in LECs. **A** Expression profiles of cytokines in NF-CM and CAF-CM. **B** Overlap between lymphangiogenesis-related and EMT-related proteins. **C** The expression of the four significant cytokines was analysed by qRT-PCR. **D** The secretion of PAI-1 from primary fibroblasts was analysed by ELISA. **E** HDLECs were treated with CM in the presence of control anti-IgG (represented as “-”; GeneTex, GTX35009, 20 μg/ml) or anti-PAI-1 antibody (represented as “ + ”; GeneTex, GTX79745, 20 μg/ml). Cell lysates were subjected to Western blotting with antibodies against the indicated proteins. **F** Representative micrographs of migration assays (upper panels), tube formation assays (intermediate panels) and transendothelial assays (lower panels) using HLECs pretreated with the indicated treatments. **G** The average number of migrated cells per field was calculated. **H** The average tube length per field was calculated. **I** The number of transmigrated SiHa-mCherry cells on the bottom side of the HDLEC monolayer was quantified. Data are presented as the mean ± SD of three independent experiments. *P < 0.05

CAFs employ a more complicated cytokine expression profile than NFs to make the TME suitable for cancer progression. To elucidate the factors involved in CAF-mediated EndoMT, we performed cytokine array analyses. A search for differentially expressed cytokines identified thirty that were upregulated in CAFs relative to NFs (fold change > 1.5) (Fig. 4A). Among these upregulated cytokines, four were annotated as lymphangiogenesis-associated proteins and EMT-related proteins (Fig. 4B), including PAI-1, FLRG, RGMB, and TGF-β. Notably, PAI-1 was validated to be the most significantly upregulated cytokine by qPCR (* *P* < 0.05) (Fig. 4C). The ELISA assay further determined that PAI-1 levels were higher in the CM of CAFs than those in the CM of NFs (* *P* < 0.05) (Fig. 4D).

To characterize the possible effect of CAF-secreted PAI-1 on the EndoMT of LECs, secreted PAI-1 in CAF-CM was immunodepleted with an anti-PAI-1 antibody, and an anti-IgG antibody (isotype control) was used as a negative control. Western blotting analysis revealed that compared with the control, the anti-PAI-1 antibody remarkably abrogated the CAF-CM-induced expression of FAP, α-SMA and vimentin and the downregulation of VE-cadherin (Fig. 4E). Correspondingly, in vitro studies confirmed that the EndoMT-related functional changes in response to CAF-CM treatment, including the increased cell migration (Fig. 4G, F), hyperpermeability (Fig. 4I, F) and tube formation (Fig. 4H, F), were suppressed by the PAI-1 blocking antibody. These results establish a critical role of PAI-1 in mediating CAF-dependent EndoMT.

### Secreted extracellular PAI-1 promotes EndoMT in LECs by activating the LRP-ERK-AKT signalling pathways

**Fig. 5:**
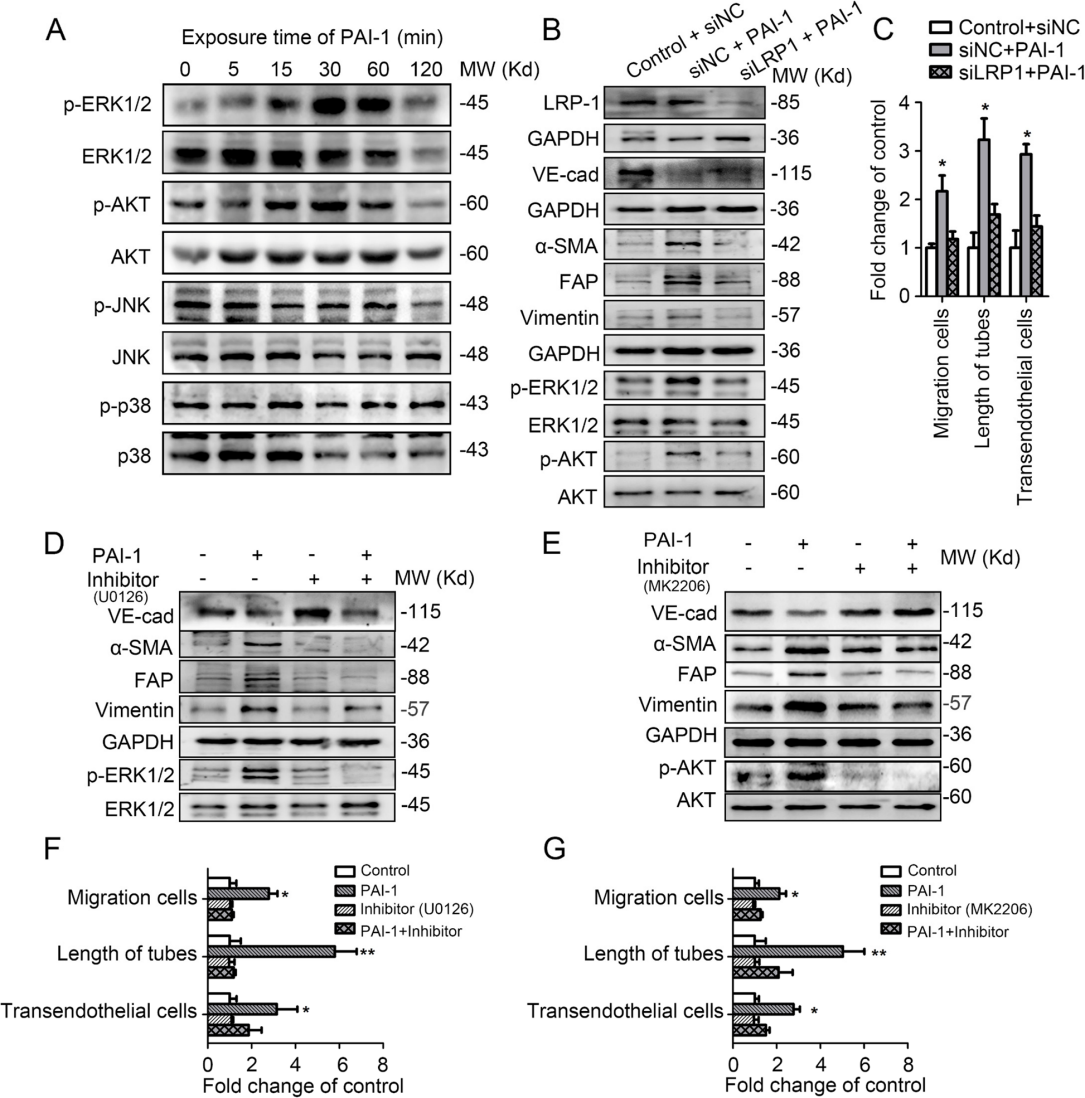
Secreted extracellular PAI-1 promotes EndoMT in LECs by activating the LRP-ERK-AKT signalling pathways. **A** Western blotting analysis of the signalling pathways activated in PAI-1-treated HDLECs. **B** HDLECs transduced with siNC or siLRP1 were treated with ECM or CM for 1 h. Cell lysates were analysed by Western blotting. **C** The migration, tube formation and transendothelial assays were analysed following the indicated treatments. **D** Western blot analysis of ERK1/2 activation and the expression levels of VE-cadherin, α-SMA, FAP, and vimentin in HDLECs treated or not with PAI-1 in the presence of control or U0126 (ERK1/2 inhibitor) for 1 h. **E** Western blot analysis of AKT activation and the expression levels of VE-cadherin, α-SMA, FAP, and vimentin in HDLECs treated or not with PAI-1 in the presence of control or MK2206 (AKT inhibitor) for 1 h. **F**. The migration, tube formation and transendothelial assays were analysed following the treatments described in **D**. **G** The migration, tube formation and transendothelial assays were analysed following the treatments described in **E** Error bars represent the mean ± SD of three independent experiments. *, *P* < 0.05; **, *P* < 0.01

PAI-1 regulates certain biological functions by binding to LRP1 to activate downstream signalling cascades in recipient cells [18]. LRP1 has been reported to mediate the activation of the MAPK signal pathway [19]. We performed western blotting to interrogate potential PAI-1-related signalling pathways, including the ERK1/2, AKT, JNK and p38 pathways. After PAI-1 treatment, peal ERK1/2 and AKT phosphorylation occurred at 30 min (Fig. 5A). Other signalling pathway indicators, including JNK and p38, were not activated up to 120 min after PAI-1 treatment. To further identify the function of ERK1/2 and AKT signalling in mediating PAI-1-induced EndoMT in HDLECs, cells were treated with specific inhibitors of ERK1/2 (U0126) and AKT (MK2206). Inhibition of ERK1/2 and AKT obviously blocked the PAI-1-induced upregulation of FAP, α-SMA and vimentin and downregulation of VE-cadherin (Fig. 5D and E), suggesting that the phosphorylation of ERK1/2 and AKT is required for PAI-1-induced EndoMT. Consistently, migration, tube formation and transendothelial migration assays demonstrated that the functional association of ERK1/2/AKT signalling with EndoMT-related lymphangiogenesis was blocked by U0126 and MK2206 (* P < 0.05, Fig. 5F and G, Figs. S3 and S4).

Because PAI-1 is a ligand for LRP1 [20], we sought to determine the relationship between PAI-1-dependent ERK1/2/AKT activation and LRP1 binding. We knocked down LRP1 expression in HDLECs using small interfering RNA (siRNA) and confirmed the effect by qPCR analysis (Fig. S5). Western blotting analysis showed that siLRP1 suppressed the PAI-1-induced ERK1/2 and AKT phosphorylation and EndoMT phenotype in HDLECs (Fig. 5B). Correspondingly, siLRP1 significantly abrogated the EndoMT-related lymphangiogenesis induced by PAI-1 (Fig. 5C, Fig. S2). Taken together, these results demonstrate that the interaction of PAI-1 with endothelial LRP1 triggers ERK1/2/AKT signalling, consequently promoting EndoMT in HDLECs and abnormal lymphangiogenesis.

### PAI-1 drives EndoMT and aberrant lymphangiogenesis and correlates with poor prognosis in CSCC

**Fig. 6:**
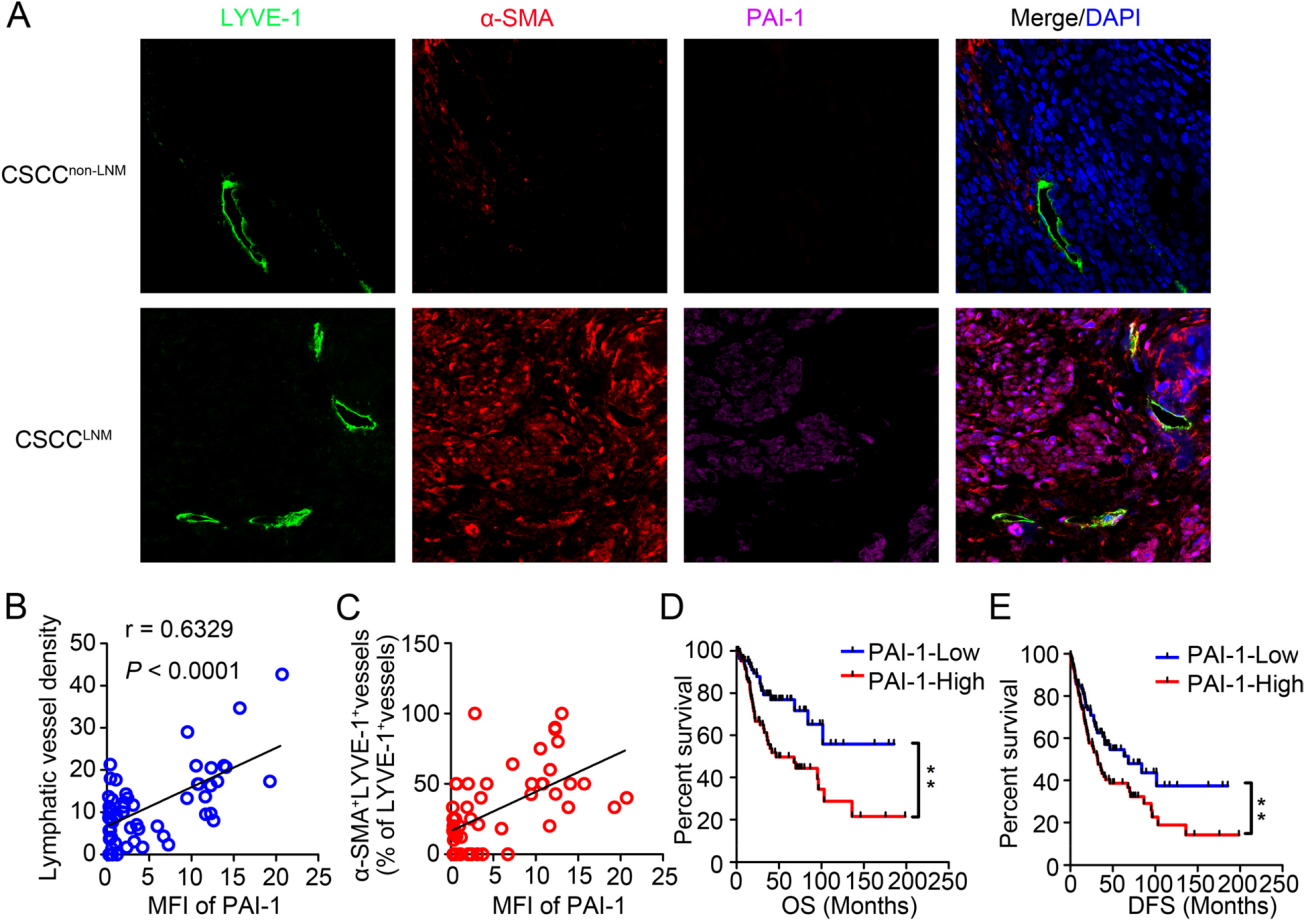
PAI-1 drives EndoMT and aberrant lymphangiogenesis and correlates with poor prognosis in CSCC. **A** Representative images of CSCC tissues stained for LYVE-1 (green), α-SMA (red), or PAI-1 (purple). Blue indicates the nucleus. **B** The correlation between PAI-1 expression level and LVD in CSCC tissues. **C** The correlation between PAI-1 expression level and the percentage of α-SMA.^+^LVs in CSCC tissues. **D** and **E** The OS (D) and DFS (E) of CSCC patients from the TCGA with lower versus higher PAI-1 expression were estimated using Kaplan–Meier curves. The median expression was used as the cut-off value. Data are presented as the mean ± SD. **P < 0.0001

To clarify the relationships among PAI-1 expression, LVD and EndoMT in CSCC patients, we used triple immunofluorescent staining for PAI-1, LYVE-1 and α-SMA in CSCC tissues. We observed colocalization of PAI-1 and α-SMA in the stromal regions of CSCC tissues, confirming that PAI-1 was preferentially expressed by CAFs. Stromal PAI-1 expression was significantly higher in CSCC^LNM^ tissues than in CSCC^non−LNM^ tissues. Moreover, stromal PAI-1 expression was positively correlated with increased LVD (*r* = 0.6329, *P* < 0.0001, Fig. 4B) and α-SMA^+^ LVs (*r* = 0.5724, *P* < 0.0001, Fig. 4C). These data suggest that CAF-derived PAI-1 is critical for EndoMT-related lymphangiogenesis in CSCC. To evaluate the relevance of PAI-1 to clinical prognosis, we analysed public data from TCGA, which revealed that increased levels of PAI-1 were associated with a shorter OS (*P* = 0.0019, log-rank test) and DFS (*P* < 0.0245, log-rank test) in CSCC (Fig. 6D and E). Taken together, our data suggest that stromal PAI-1 may be a diagnostic biomarker and therapeutic target for CSCC.

**Fig. 7:**
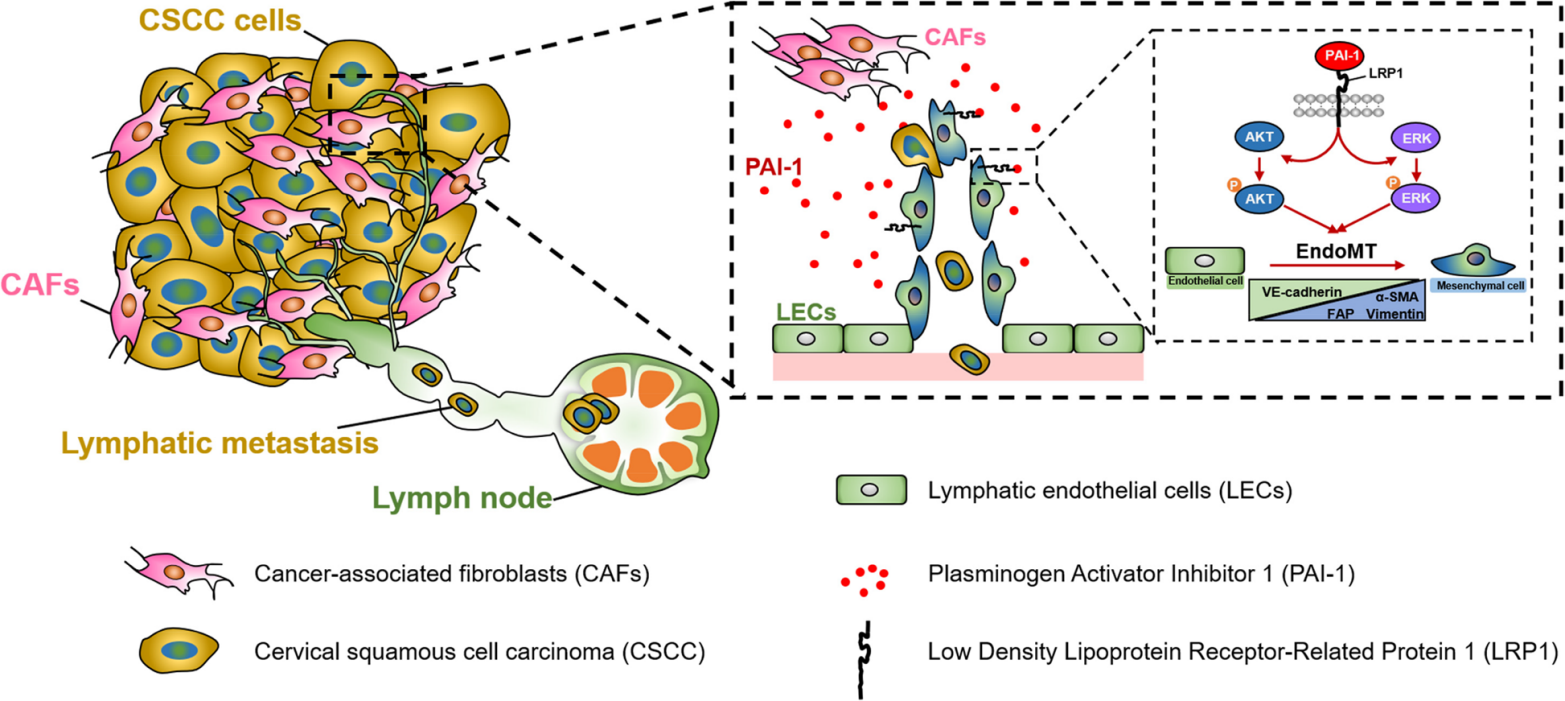
Schematic model. CAF-derived PAI-1 induces EndoMT in tumour-associated LECs by activating the LRP1/ERK1/2/AKT signalling pathway, which in turn drives abnormal lymphangiogenesis by increasing LEC migration, tube formation and monolayer permeability, thereby facilitating tumour lymphoinvasion and CSCC progression

Collectively, our findings indicated that CAF-derived PAI-1 induces EndoMT in LECs by activating the LRP1/ERK1/2/AKT pathway, which in turn drives abnormal lymphangiogenesis, thereby facilitating tumour lymphoinvasion and CSCC progression. The mechanisms presented in this study are summarized in a schematic diagram (Fig. 7).

## Discussion

Lymphatic remodelling in advance of lymphatic metastasis represents the formation of a prometastatic TME [1], but the underlying dynamics and mechanisms have not yet been studied in detail. EndoMT is characterized as the cellular transition from an endothelial to a mesenchymal phenotype, which is an important process in lymphatic remodelling [21]. In this study, our clinical evidence showed the occurrence of EndoMT in LECs in CSCC, which was positively correlated with lymphatic metastasis, suggesting that lymphatic remodelling through EndoMT could influence CSCC metastasis. Recently, Huang et al. [7] revealed that tumour-associated endothelial cells undergo EndoMT without losing endothelial functions, leading to aberrant tumour vascularization in glioblastoma multiforme. To the same extent, EndoMT in LECs stimulated by Wnt ligand has been implicated in oral cancer lymphangiogenesis [9]. In contrast with its role in the de novo generation of fibroblasts, these findings highlight a major function of EndoMT in lymphovascular regulation [22]. Consistently, our data showed that highly aggressive EndoMT is closely correlated with LVD, suggesting that LEC EndoMT may be important for the extensive lymphovascularity observed in the prometastatic niche in CSCC.

The TME is a dynamic system orchestrated by intercellular crosstalk that contributes to the regulation of EndoMT in LECs. CAFs, the most abundant stromal cells in the TME, have been the focus of studies since they not only provide physical support for epithelial cells but also are key functional regulators in cancer. Herein, we revealed the existence of a dynamic association between LECs undergoing EndoMT and adjacent CAFs. CAFs promoted EndoMT in LECs, including downregulating VE-cadherin and upregulating α-SMA, vimentin and FAP, accompanied by EndoMT-related lymphangiogenesis. Due to the loss of VE-cadherin and the reorganization of the endothelial cytoskeleton, EndoMT disrupts the endothelial barrier to favour tumour transendothelial migration. We demonstrated that EndoMT-related lymphatics induced by CAFs actively encouraged metastatic intravasation in CSCC, indicating an essential role of CAF-induced EndoMT in optimal metastatic dissemination in CSCC.

CAFs regulate cellular function via their active secretome, and CAFs from different cancers have distinct cytokine expression patterns. We found that PAI-1 was significantly upregulated in CAFs from CSCC. It is well known that PAI-1 can neutralize the activities of tPA and uPA to impede plasmin formation and protect ECM proteins from proteolytic degradation [23]. Therefore, a sustained increase in PAI-1 levels may result in collagen accumulation and thus tissue remodelling [24]. Recently, PAI-1 has also been linked to cancer. According to Masuda et al. [25], inhibiting PAI-1 suppressed the MF characteristics of CAFs and limited chemotherapy resistance in lung cancer. Furthermore, the absence of PAI-1 in transgenic mice decreased tumour growth and angiogenesis [26]. Our findings showed that LECs treated with PAI-1 exhibited a higher level of the mesenchymal phenotype. Blockade of PAI-1 resulted in the abrogation of CAF-mediated LEC EndoMT and further inhibited EndoMT-related lymphangiogenesis. Interestingly, an increasing number of CAFs was also observed in the CAF-CM treated group. In conjunction with prior research [25, 27], we considered that the positive feedback effect of PAI-1 in CAFs promoted the occurrence of EndoMT even further. Moreover, clinical evidence combined with public data indicated that high PAI-1 expression was positively correlated with lymphatic metastasis, poor OS and DFS. These results revealed that CAFs with elevated PAI-1 expression form a favourable niche for LEC EndoMT and promote the metastatic progression of CSCC, which might explain the poor outcome of these patients. Although PAI-1 has been reported to promote tumour progression in some studies, we report its novel function in mediating EndoMT during crosstalk between CAFs and LECs.

We then examined the regulatory mechanisms of PAI-1-mediated EndoMT in CSCC. The pleiotropic biological function of PAI-1 stems from its complex structure [28]. The ability of PAI-1 to bind to LRP1 directly leads to intracellular signalling and cell migration [29]. In this study, PAI-1-dependent EndoMT in LECs was suppressed by knocking down LRP1 expression in LECs, indicating that the interaction between LRP1 and deposited PAI-1 is critical for promoting LEC transdifferentiation. ERK1/2 and AKT signalling have been recognized as downstream survival signals that regulate LRP1-dependent cell functions [30]. Recently, the important roles of these signalling pathways in inducing the mesenchymal phenotype in endothelial or epithelial cells has been highlighted in various cancers [31]. We observed that in LECs cultured with PAI-1, the levels of phosphorylated ERK1/2 and AKT were selectively decreased in LRP1-knockdown LECs, suggesting that LRP1 is required for the activation of ERK1/2 and AKT signalling in PAI-1-treated LECs. Furthermore, the inhibition of ERK1/2 or AKT blocked the dysregulated expression of genes related to the mesenchymal phenotype and further abrogated EndoMT-related lymphangiogenesis in PAI-1-treated LECs, which verified the important role of these proteins in regulating LEC activities related to EndoMT. Taken together, the data suggest that the constant accumulation of CAF-derived PAI-1 in the TME induces ERK1/2 and AKT phosphorylation by binding to LRP1 in recipient cells. Phosphorylated ERK1/2/AKT further initiates the EndoMT programme in LECs, resulting in extensive lymphovascularity in CSCC.

Lymphatic metastasis is the principal reason for the poor survival rate of CSCC patients. A detailed understanding of how LVs contribute to metastasis is crucial to better comprehend the biological complexity of lymphatic metastasis. EndoMT is a developmental programme that recapitulates neolymphangiogenesis in response to the TME. Inhibiting EndoMT by blocking regulatory pathways or by promoting its reverse programme offers a new paradigm for antimetastatic therapy. In this study, the PAI-1-mediated EndoMT of LECs stimulated migration, hyperpermeability and tube formation, thus causing aberrant lymphangiogenesis and promoting lymphatic metastasis in CSCC. As such, the targeted ablation of PAI-1 in CAFs or selected knockdown of LRP1 in LECs could suppress EndoMT-related lymphangiogenesis. Characterization of LEC EndoMT and the regulatory mechanisms will offer novel targets for the development of antimetastatic therapy and provide a basis for the selection of specific cohorts of patients who might benefit from certain molecular-targeted drugs.

## Supplementary Information

**Additional file 1: Figure S1.** HDLEC monolayers incubated with the indicated CM for 24 h were analysed by immunofluorescence (IF) staining of VE-cadherin (red). Blue indicates the nucleus. **Figure S2.** siLRP1 abolished the biological effects of PAI-1 on HDLECs, related to Fig 5C. **Figure S3.** Suppression of ERK1/2 phosphorylation by U0126 in HDLECs affected the biological behaviour of PAI-1, related to Fig 5F. **Figure S4.** Suppression of AKT phosphorylation by MK2206 in HDLECs affected the biological behaviour of PAI-1, related to Fig 5G. **Figure S5.** Validation and efficacy of LRP1 gene silencing was performed by qPCR. Normalized gene expression over GAPDH is shown with the means ± SD of three independent experiments. *P < 0.05.

**Additional file 2: Table S1.** Effect of CAFs on popliteal lymph nodes (LNs) metastasis in vivo. **Table S2.** Primers for real-time RT-PCR.

## Data Availability

All data that can prove the conclusion of this article are included in the article.

## Abbreviations

CAFs: Cancer-associated fibroblasts
NFs: Normal fibroblasts
CSCC: Cervical squamous cell carcinoma
EndoMT: Endothelial–mesenchymal transition
TME: Tumour microenvironment
LVs: Lymphatic vessels
LECs: Lymphatic endothelial cells
HDLECs: Human dermal lymphatic endothelial cells
CM: Conditioned medium
siRNA: Small interfering RNA
TCGA: The Cancer Genome Atlas
α-SMA: Alpha smooth muscle actin
FAP: Fibroblast activated protein
