# Supplementary material for: Cancer-associated fibroblast-derived PAI-1 promotes lymphatic metastasis via the induction of EndoMT in lymphatic endothelial cells
Source: J Exp Clin Cancer Res. 2023 Jul 6;42:160. doi: 10.1186/s13046-023-02714-0 (PMC10324144; doi:10.1186/s13046-023-02714-0)

Supplementary Figures:

Figure S1. HDLEC monolayers incubated with the indicated CM for 24 h were analysed by immunofluorescence (IF) staining of VE-cadherin (red). Blue indicates the nucleus.

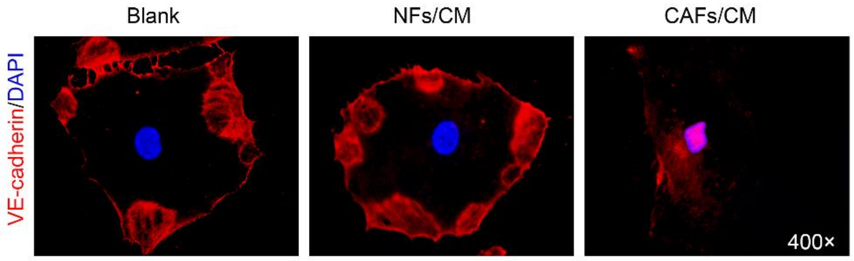

Figure S2. siLRP1 abolished the biological effects of PAI-1 on HDLECs, related to Fig 5C. **A.** Representative micrographs of the Transwell assay with HDLECs. **B.** Representative micrographs of the tube formation assay. Scale bar, 50 μm. **C.** HDLEC monolayer following the indicated treatments described in **Fig. 5C** and quantification of SiHa-mCherry cell migration.

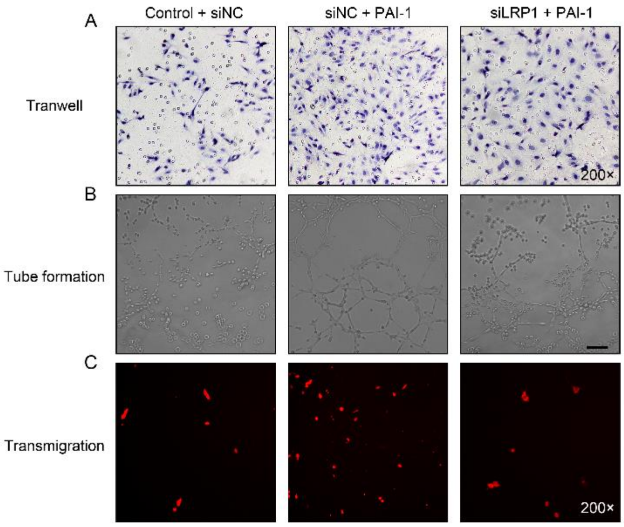

Fig S3. Suppression of ERK1/2 phosphorylation by U0126 in HDLECs affected the biological behaviour of PAI-1, related to Fig 5F.

A. Representative micrographs of the Transwell assay in HDLECs. **B.** Representative micrographs of the tube formation assay. Scale bar, 50 μm. **C.** HDLEC monolayer following the indicated treatments described in **Fig. 5G** and quantification of SiHa-mCherry cell migration.

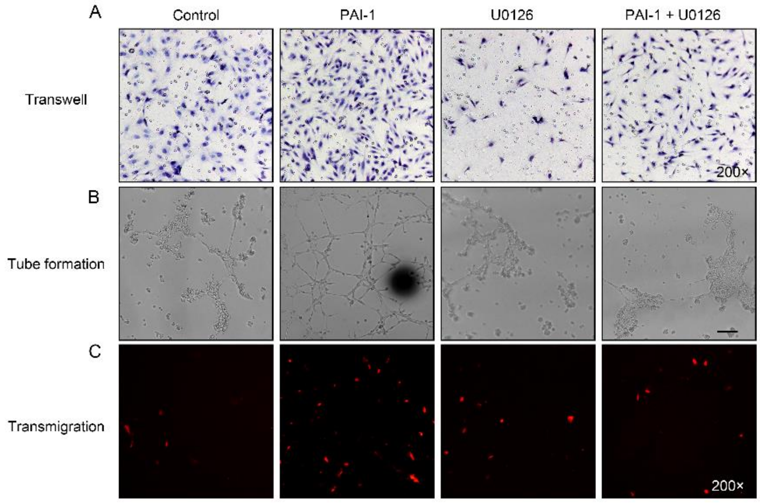

Fig S4. Suppression of AKT phosphorylation by MK2206 in HDLECs affected the biological behaviour of PAI-1, related to Fig 5G.

A. Representative micrographs of the Transwell assay with HDLECs. **B.** Representative micrographs of the tube formation assay. Scale bar, 50 μm. **C.** HDLEC monolayer following the indicated treatments described in **Fig. 5F** and quantification of SiHa-mCherry cell migration.

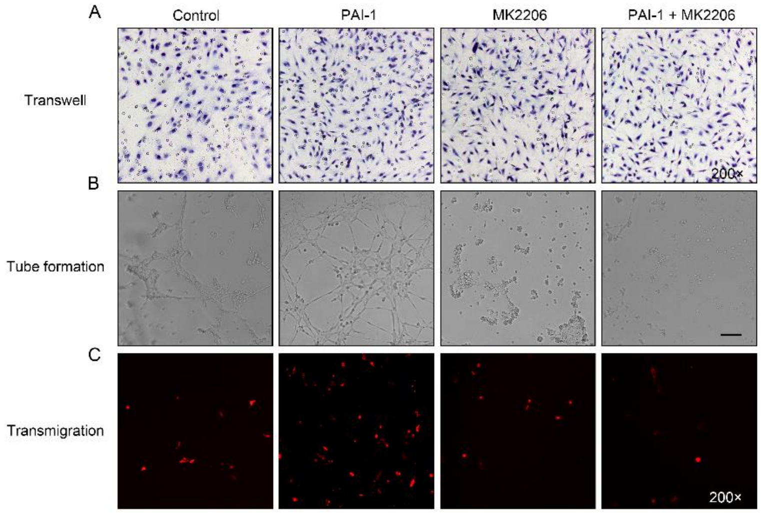

Fig S5. Validation and efficacy of LRP1 gene silencing was performed by qPCR. Normalized gene expression over GAPDH is shown with the means ± SD of three

independent experiments. *P < 0.05.

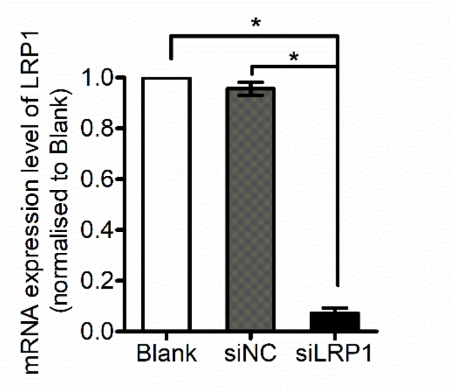

Supplement: Supplementary file 1 — Additional file 1: Figure S1. HDLEC monolayers incubated with the indicated CM for 24 h were analysed by immunofluorescence (IF) staining of VE-cadherin (red). Blue indicates the nucleus. Figure S2. siLRP1 abolished the biological effects of PAI-1 on HDLECs, related to Fig 5C. Figure S3. Suppression of ERK1/2 phosphorylation by U0126 in HDLECs affected the biological behaviour of PAI-1, related to Fig 5F. Figure S4. Suppression of AKT phosphorylation by MK2206 in HDLECs affected the biological behaviour of PAI-1, related to Fig 5G. Figure S5. Validation and efficacy of LRP1 gene silencing was performed by qPCR. Normalized gene expression over GAPDH is shown with the means ± SD of three independent experiments. *P < 0.05. [file 13046_2023_2714_MOESM1_ESM.pdf]
