# Supplementary material for: Cancer-associated fibroblast-derived PAI-1 promotes lymphatic metastasis via the induction of EndoMT in lymphatic endothelial cells
Source: J Exp Clin Cancer Res. 2023 Jul 6;42:160. doi: 10.1186/s13046-023-02714-0 (PMC10324144; doi:10.1186/s13046-023-02714-0)
Supplement: Supplementary file 2 — Additional file 2: Table S1. Effect of CAFs on popliteal lymph nodes (LNs) metastasis in vivo. Table S2. Primers for real-time RT-PCR. [file 13046_2023_2714_MOESM2_ESM.pdf]

Table S1. Effect of CAFs on popliteal lymph nodes (LNs) metastasis *in vivo*.

	NO. total LNs	NO. positive LNs	Positive ratio (%)
Control	10	3	30%
NFs/CM	10	5	30%
CAFs/CM	10	7	70%

Table S2. Primers for real-time RT-PCR.

Gene	PCR primers (5'-3')	
	Sense primers	Antisense primers
GAPDH	TGCACCACCAACTGCTTAGC	GGCATGGACTGTGGTCATGAG
PAI-1	AGTGGACTTTTCAGAGGTGGA	GCCGTTGAAGTAGAGGGCATT
FLGR	TGCTCAGAATCGCCTACC	CTCCGTGTTGTCCTCTCC
RGM-B	TGTTGGGTATCAGTGACCTCA	CTTCGGGGTTGGTAGAGGATG
TGFβ1	CTAATGGTGGAAACCCACAACG	TATCGCCAGGAATTGTTGCTG
